# Indoxyl Sulfate, Cardiac Biomarkers, and Left Ventricular Wall Thickening in Cats with Chronic Kidney Disease

**DOI:** 10.3390/vetsci13090989

**Published:** 2026-09-19

**Authors:** Murat Akbaba, Idil Bastan, Filiz Bakar-Ates, Osman Safa Terzi

**Affiliations:** 1 Department of Internal Medicine, Faculty of Veterinary Medicine, Ankara University, 06070 Ankara, Turkey; 2 Department of Biochemistry, Faculty of Pharmacy, Ankara University, 06560 Ankara, Turkey; fbakar@ankara.edu.tr

**Keywords:** feline chronic kidney disease, left ventricular wall thickening, indoxyl sulfate, p-cresyl sulfate, NT-proBNP, cardiac troponin I, systolic hypertension

## Abstract

Factors associated with left ventricular wall thickening (LVWT) in cats with chronic kidney disease (CKD) remain unclear. This prospective observational study compared renal variables, systolic blood pressure (SBP), echocardiographic measurements, cardiac biomarkers, and uremic toxins in 31 cats: 12 with CKD without LVWT, 8 with CKD and LVWT, and 11 healthy controls. LVWT was defined as IVSd and/or LVPWd ≥ 6 mm in at least two of three examinations. IRIS stage distribution did not differ between the CKD groups (*p* = 1.000). Urea, creatinine, and phosphorus concentrations were higher in both CKD groups than in controls but were similar between CKD groups. SBP, NT-proBNP, and cardiac troponin I concentrations were highest in the CKD-LVWT group (*p* < 0.001). Plasma indoxyl sulfate concentrations were significantly higher in the CKD-LVWT group than in both other groups, whereas serum p-cresyl sulfate concentrations did not differ between CKD groups. However, SBP was markedly higher in the CKD-LVWT group and represents a major potential confounder. However, because SBP was also higher in the CKD-LVWT group and reliable multivariable adjustment could not be performed, the independent contribution of indoxyl sulfate could not be determined. The observed association may have been influenced by systemic hypertension, CKD stage, antihypertensive treatment and adherence, and other potential confounders and should not be interpreted as independent or causal.

## Simple Summary

Chronic kidney disease (CKD) is common in older cats and may also affect the cardiovascular system. We evaluated 31 cats: 12 with CKD without left ventricular wall thickening (LVWT), 8 with CKD and LVWT, and 11 healthy controls. Blood pressure, echocardiographic measurements, cardiac biomarkers, indoxyl sulfate, and p-cresyl sulfate were assessed. Cats with CKD and LVWT had the highest systolic blood pressure and concentrations of NT-proBNP, cardiac troponin I, and indoxyl sulfate. Urea, creatinine, and phosphorus concentrations were similarly increased in both CKD groups. P-cresyl sulfate concentrations were also increased in both CKD groups but did not differ according to LVWT status. However, systolic blood pressure was highest in the CKD-LVWT group and represents a major potential confounder. Therefore, the observed association between indoxyl sulfate and LVWT cannot be considered independent of blood pressure. These findings are exploratory and require confirmation in larger longitudinal studies.

## 1. Introduction

Chronic kidney disease (CKD) is one of the most common disorders affecting older cats [1,2,3] and may be accompanied by systemic and cardiovascular complications [4]. As renal function declines, disturbances in fluid balance, phosphorus homeostasis, blood pressure regulation, and the elimination of uremic solutes may contribute to cardiovascular dysfunction [5]. Systolic hypertension is a well-recognized complication of feline CKD and can result in target-organ injury, including alterations in myocardial structure and increased left ventricular wall thickness [6,7]. However, the relative contributions of hemodynamic, metabolic, and uremia-related factors to cardiac structural alterations in cats with CKD remain incompletely understood [7,8].

Cardiac alterations associated with CKD may include increased left ventricular wall thickness and impaired diastolic function [6]. These abnormalities may develop in response to chronic pressure overload, volume-related stress, metabolic disturbances, or direct uremia-associated myocardial injury. Conventional renal variables, including serum urea, creatinine, and phosphorus concentrations, are useful for assessing the severity of renal dysfunction but do not directly reflect the extent of concurrent myocardial stress or injury [5].

N-terminal pro-B-type natriuretic peptide (NT-proBNP) and cardiac troponin I (cTnI) provide complementary information regarding cardiac stress and injury. NT-proBNP is released in response to myocardial stretch caused by pressure or volume overload, whereas cTnI is a marker of cardiomyocyte injury. Both biomarkers are used to assess cardiac disease in cats for the assessment of cardiac disease [9,10]. However, their interpretation may be complicated by renal dysfunction. Reduced renal clearance, particularly of natriuretic peptides, together with subclinical volume overload and uremia-associated myocardial injury may result in increased biomarker concentrations even in the absence of overt structural heart disease [4,7,8,11]. Therefore, these biomarkers should be interpreted in conjunction with blood pressure and echocardiographic findings in cats with CKD.

Protein-bound uremic toxins represent another potential link between renal dysfunction and cardiovascular injury. Indoxyl sulfate (IS) and p-cresyl sulfate (PCS) are gut-derived uremic retention solutes that accumulate as renal excretory function declines [12,13]. Experimental studies indicate that IS may promote several processes involved in cardiovascular remodeling, including oxidative stress, inflammation, myocardial fibrosis, cardiomyocyte hypertrophy, endothelial dysfunction, and increased vascular stiffness [14,15,16,17,18]. To the authors’ knowledge, the relationships among indoxyl sulfate, p-cresyl sulfate, and echocardiographic evidence of left ventricular wall thickening (LVWT) have not previously been investigated in cats with naturally occurring CKD.

Therefore, this study aimed to compare renal variables, systolic blood pressure, echocardiographic findings, cardiac biomarkers (NT-proBNP and cTnI), and protein-bound uremic toxin concentrations (plasma indoxyl sulfate and serum p-cresyl sulfate) among healthy controls, cats with IRIS stage 3 or 4 CKD without LVWT, and cats with IRIS stage 3 or 4 CKD and LVWT. We hypothesized that cats in the CKD-LVWT group would have higher systolic blood pressure and higher concentrations of cardiac biomarkers, plasma ID, and serum PCS than the other two groups.

## 2. Materials and Methods

### 2.1. Animal Population

This study was conducted at the Animal Hospital of the Faculty of Veterinary Medicine, Ankara University, between March 2021 and March 2023. The study population consisted of client-owned cats diagnosed with chronic kidney disease and clinically healthy control cats. A total of 31 cats were included. The study protocol was approved by the Institutional Animal Care and Use Committee of Ankara University (Approval No: 2021-15-137).

Cats were allocated into three groups according to renal status and echocardiographic evidence of LVWT: CKD without LVWT (CKD-NoLVWT; *n* = 12), CKD with left ventricular wall thickening (CKD-LVWT; *n* = 8), and clinically healthy controls (control; *n* = 11). The control group included cats with no clinical, hematological, biochemical, or echocardiographic evidence of renal or cardiac disease. Both CKD groups included cats classified as IRIS stage 3 or 4, according to the 2023 IRIS CKD staging guidelines [19].

The CKD-noLVWT group comprised three Persian cats, one Scottish Fold, one Turkish Angora, one British Shorthair, and six mixed-breed cats. The CKD-LVWT group comprised one Persian, one Scottish Fold, one Turkish Angora, and five mixed-breed cats. The control group comprised two Persian cats, four Scottish Fold cats, one Turkish Angora, two British Shorthair cats, and two mixed-breed cats.

Chronic kidney disease was diagnosed on persistent renal azotemia, defined as serum urea and creatinine concentrations remaining above the laboratory reference intervals on repeated evaluations over a period of at least three months, together with inadequate urine-concentrating ability, defined as a urine specific gravity (USG) < 1.035. USG was measured using a feline-specific handheld digital refractometer (PAL-USG [CAT], ATAGO Co., Ltd., Tokyo, Japan). Proteinuria substaging was not performed because urine protein-to-creatinine ratio measurements were not available for the study population. First-morning urine samples were collected from all cats by cystocentesis and used for urinalysis and USG measurement. Following diagnosis, cats were staged according to the IRIS CKD staging guidelines using serum creatinine concentrations obtained when they were clinically stable. Only cats classified as IRIS stage 3 or 4 were included. The CKD-noLVWT group comprised four cats with IRIS stage 3 CKD and eight with stage 4 CKD, whereas the CKD-LVWT group comprised two cats with stage 3 CKD and six with stage 4 CKD.

All cats underwent physical examination, systolic blood pressure measurement, transthoracic echocardiography, and routine hematological and serum biochemical analyses. Cats with hyperthyroidism, neoplasia, congenital cardiac disease, clinically relevant valvular disease, pericardial effusion, or any concurrent systemic disorder that could independently influence renal or cardiac function were excluded.

Cats with CKD received supportive treatment according to their individual clinical requirements. Amlodipine was prescribed to hypertensive cats in accordance with the ISFM consensus guidelines [6], and the prescribed dose was individually determined on the basis of SBP. In the CKD-noLVWT group, 3 of 12 cats were hypertensive and had been prescribed amlodipine; their owners reported inconsistent administration. In the CKD-LVWT group, 6 of 8 cats were hypertensive and had been prescribed amlodipine; however, their owners reported that the prescribed medication had never been administered. The remaining 2 cats in the CKD-LVWT group were normotensive and had not been prescribed amlodipine. No control cats received amlodipine. Antihypertensive treatment was prescribed for clinical reasons and was not assigned or withheld as part of the study. Treatment and adherence status were not used for group allocation.

Because no previous feline studies had compared indoxyl sulfate concentrations according to the presence of LVWT in cats with CKD, sufficient preliminary data were unavailable for a reliable a priori sample-size calculation. Therefore, this study was designed as an exploratory investigation, and all cats meeting the eligibility criteria during the study period were included.

### 2.2. Blood Pressure Measurement

Systolic blood pressure (SBP) was measured non-invasively using a Vet BP Doppler ultrasonic flow detector (Burtons Medical Equipment Ltd., Marden, UK) in accordance with ACVIM guidelines [20]. Measurements were obtained in a quiet environment after a short acclimatization period. The first reading was discarded, and the mean of five consecutive measurements was used for statistical analysis. Systemic hypertension was defined as SBP ≥ 160 mmHg on at least two separate occasions.

### 2.3. Echocardiographic Examination

Transthoracic echocardiographic examinations were performed using a Hitachi Arietta V60 ultrasound system (Hitachi Aloka Medical Ltd., Mitaka, Tokyo, Japan) equipped with a 2–9 MHz phased-array transducer. Cats were examined without sedation and gently restrained in right and left lateral recumbency. Standard right parasternal long-axis and short-axis views were obtained.

Left ventricular chamber dimensions, interventricular septal thickness in diastole (IVSd), and left ventricular posterior wall thickness in diastole (LVPWd) were measured using the leading-edge-to-leading-edge method. For each examination, measurements were obtained from at least three technically adequate consecutive cardiac cycles, and the mean value was recorded. Left atrial size was assessed using the left atrial-to-aortic root ratio (LA/Ao) obtained from the right parasternal short-axis view at the level of the aortic valve. Left ventricular ejection fraction (EF) was estimated from M-mode-derived linear dimensions using the Teichholz method.

Available medical and echocardiographic records were reviewed before enrollment to identify cats with previously diagnosed overt primary cardiac disease. Cats with a previous diagnosis of primary cardiomyopathy or overt echocardiographic features suggestive of an HCM phenotype, other than increased LV wall thickness alone, were excluded. These features included marked or regionally asymmetric LV hypertrophy, systolic anterior motion of the mitral valve, dynamic left ventricular outflow tract obstruction, and prominent papillary muscle hypertrophy. Cats with congenital cardiac disease, clinically relevant valvular disease, systolic dysfunction, or pericardial effusion were also excluded.

Echocardiographic examinations were repeated three times within a one-month period. For the purposes of this study, LVWT was defined as an IVSd and/or LVPWd ≥ 6 mm in at least two of the three examinations. Cats with CKD were subsequently classified as CKD-LVWT or CKD-noLVWT according to this echocardiographic phenotype. This classification was based on the presence of CKD and LVWT and was not intended to establish the underlying etiology of the wall thickening or to definitively exclude occult or early-stage primary HCM. For comparisons of continuous echocardiographic variables, measurements obtained during the examination closest to the date of blood sampling were used.

### 2.4. Sample Collection

Blood samples were collected from all cats on the same day as the echocardiographic examination used for the primary analysis. Samples intended for hematological and biochemical analyses were processed immediately. Hematological analyses were performed at the Clinical Diagnostic Laboratory of Veterinary Medicine, Ankara University, using an automated veterinary hematology analyzer (Exigo Eos Vet, Boule Medical AB, Spånga, Sweden). Serum urea, creatinine, and phosphorus concentrations were measured using an automated clinical chemistry analyzer (RX Monaco, Randox Laboratories Ltd., Crumlin, UK).

Serum samples intended for PCS analysis and plasma samples intended for IS analysis were separated by centrifugation and stored at −80 °C until analysis. Serum NT-proBNP and cTnI concentrations were measured using feline-specific quantitative fluorescence immunoassays (Vcheck Feline NT-proBNP and Vcheck Feline Tn-I) on a Vcheck 200 analyzer (Bionote Inc., Hwaseong-si, Republic of Korea), according to the manufacturer’s instructions. Results were expressed as pmol/L for NT-proBNP and ng/mL for cTnI. The analytical measurement range of the feline NT-proBNP assay was 50–1500 pmol/L. Results above and below this range were reported as >1500 and <50 pmol/L, respectively, and were considered right- and left-censored. For statistical analyses and graphical presentation, these results were assigned values of 1500 and 50 pmol/L, respectively.

Laboratory samples were labeled with coded identifiers. The investigator performing the indoxyl sulfate and p-cresyl sulfate measurements was blinded to cats’ identities and echocardiographic group assignments. Personnel performing the routine hematological and serum biochemical analyses were also unaware of the echocardiographic findings. The investigator performing the echocardiographic examinations was blinded to the cardiac biomarker, hematological, serum biochemical, and uremic toxin results at the time of image acquisition and interpretation.

### 2.5. Uremic Toxin Analysis

Serum PCS and plasma IS concentrations were measured at the Department of Biochemistry, Faculty of Pharmacy, Ankara University. PCS and IS analytical standards were purchased from Sigma-Aldrich (St. Louis, MO, USA). Samples stored at −80 °C were thawed at room temperature immediately before analysis and prepared for chromatographic measurement. IS and PCS concentrations were determined using high-performance liquid chromatography (HPLC).

For IS analysis, 250 µL of plasma was transferred into a tube, and 900 µL of precipitation solution containing methyl paraben in acetonitrile at a concentration of 10 ng/mL was added. The mixture was vortexed for 30 s and centrifuged at 10,000 rpm for 5 min. The resulting supernatant was collected for chromatographic analysis.

For PCS analysis, serum samples were diluted 1:1 (*v*/*v*) with distilled water, followed by the addition of perchloric acid to a final concentration of 3.3%. The samples were centrifuged at 12,000× *g* for 3 min, and the resulting supernatants were collected for chromatographic analysis.

Chromatographic analyses were performed using an Agilent 1100 Series HPLC system (Agilent Technologies, Waldbronn, Germany) equipped with a diode-array detector. Separation was achieved using an ACE C18 column (250 mm × 4.6 mm, 5 µm; Advanced Chromatography Technologies Ltd., Aberdeen, UK).

For IS determination, the mobile phase consisted of ammonium hydroxide solution adjusted to pH 4.0 with formic acid and acetonitrile at a ratio of 80:20 (*v*/*v*). The flow rate was maintained at 1.0 mL/min, the column temperature was set at 35 °C, and detection was performed at 280 nm.

For PCS determination, mobile phase A consisted of 50 mM KH_2_PO_4_ buffer adjusted to pH 3.0 and acetonitrile at a ratio of 95:5 (*v*/*v*), whereas mobile phase B consisted of 50 mM KH_2_PO_4_ buffer adjusted to pH 3.0, methanol, and acetonitrile at a ratio of 1:1:1 (*v*/*v*/*v*). The flow rate was maintained at 1.0 mL/min, and detection was performed at 220 nm (Supplementary Figure S1).

The analytical methods were validated with respect to linearity, analytical range, sensitivity, accuracy, and precision. Calibration curves were generated using serial dilutions of the corresponding analytical standards, with validated ranges of 2–50 µM for IS and 5–500 µM for PCS. Linearity was assessed by linear regression analysis. Analytical sensitivity was characterized by determination of the limit of detection (LOD) and lower limit of quantification (LLOQ). Accuracy was evaluated by recovery analysis, whereas intra-day and inter-day precision were expressed as coefficients of variation (%CV). The principal analytical validation characteristics are summarized in Supplementary Table S1. The validated methods were subsequently applied to the study samples, and IS and PCS concentrations were calculated from the respective calibration curves and expressed as ng/mL.

### 2.6. Statistical Analysis

Statistical analyses were performed using IBM SPSS Statistics for Windows, version 29.0 (IBM Corp., Armonk, NY, USA). Additional post hoc and effect-size analyses were performed using Python version 3.12.14 and SciPy version 1.17.0. Continuous variables were summarized as mean ± standard deviation and median (minimum–maximum). The distribution of continuous variables was assessed using the Shapiro–Wilk test, and homogeneity of variance was evaluated using Levene’s test. Variables meeting the assumptions of parametric analysis were compared among the three groups using one-way analysis of variance, followed by Tukey’s post hoc test. Variables that did not meet these assumptions were analyzed using the Kruskal–Wallis test. When the overall Kruskal–Wallis test was significant, pairwise comparisons were performed using the Conover–Iman post hoc test with Holm adjustment for multiple comparisons. Sex distribution was compared among the groups using Pearson’s chi-square test, whereas IRIS stage distribution was compared between the two CKD groups using Fisher’s exact test. For the principal comparisons between the CKD-LVWT and CKD-noLVWT groups, effect sizes were expressed as Cliff’s delta (δ), with bias-corrected and accelerated bootstrap 95% confidence intervals based on 1,000,000 resamples. All tests were two-sided, and a *p*-value < 0.05 was considered statistically significant. In the tables, different superscript lowercase letters indicate significant pairwise differences between groups.

## 3. Results

A total of 31 cats were included: 12 cats in the CKD-noLVWT group, 8 cats in the CKD-LVWT group, and 11 in the control group. No significant differences were observed among the study groups in age or sex distribution (*p* = 0.934 and *p* = 0.726, respectively). Body weight did not differ significantly among the CKD-noLVWT, CKD-LVWT, and control groups (4.06 ± 0.48, 3.90 ± 0.46, and 4.16 ± 0.55 kg, respectively; *p* = 0.532). IRIS stage distribution was similar between the CKD groups: 4 of 12 cats in the CKD-noLVWT group and 2 of 8 cats in the CKD-LVWT group were classified as IRIS stage 3, whereas 8 and 6 cats, respectively, were classified as IRIS stage 4 (Fisher’s exact test, *p* = 1.000; Table 1).

Serum urea, creatinine, and phosphorus concentrations were significantly higher in both CKD groups than in healthy controls (*p* < 0.001; Table 2); however, none of these variables differed significantly between the CKD-noLVWT and CKD-LVWT groups.

**Figure 1:**
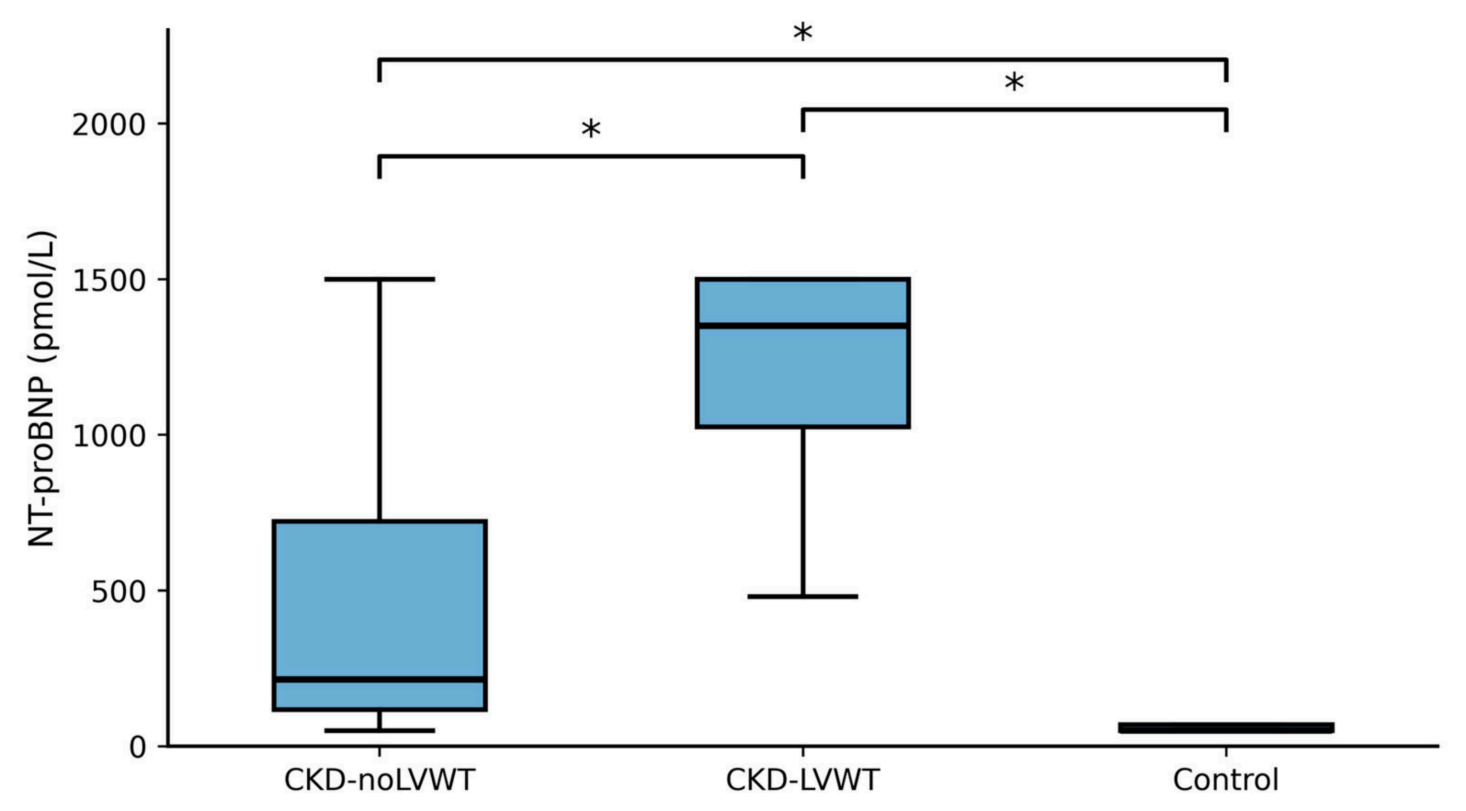
Box-and-whisker plot of serum NT-proBNP concentrations in the CKD-noLVWT group (*n* = 12), CKD-LVWT group (*n* = 8), and CKD-noLVWT group (*n* = 11). Boxes represent the interquartile range, horizontal lines within the boxes indicate the median, and whiskers extend to the most extreme values within 1.5 times the interquartile range. The overall difference among the groups was significant (Kruskal–Wallis test, *p* < 0.001). Asterisks above the horizontal brackets indicate significant pairwise differences determined using the Conover–Iman post hoc test with Holm adjustment (CKD-LVWT versus CKD-noLVWT, adjusted *p* = 0.005; CKD-noLVWT versus control and CKD-LVWT versus control, adjusted *p* < 0.001). CKD, chronic kidney disease; LVWT, left ventricular wall thickening; NT-proBNP, N-terminal pro-B-type natriuretic peptide.

**Figure 2:**
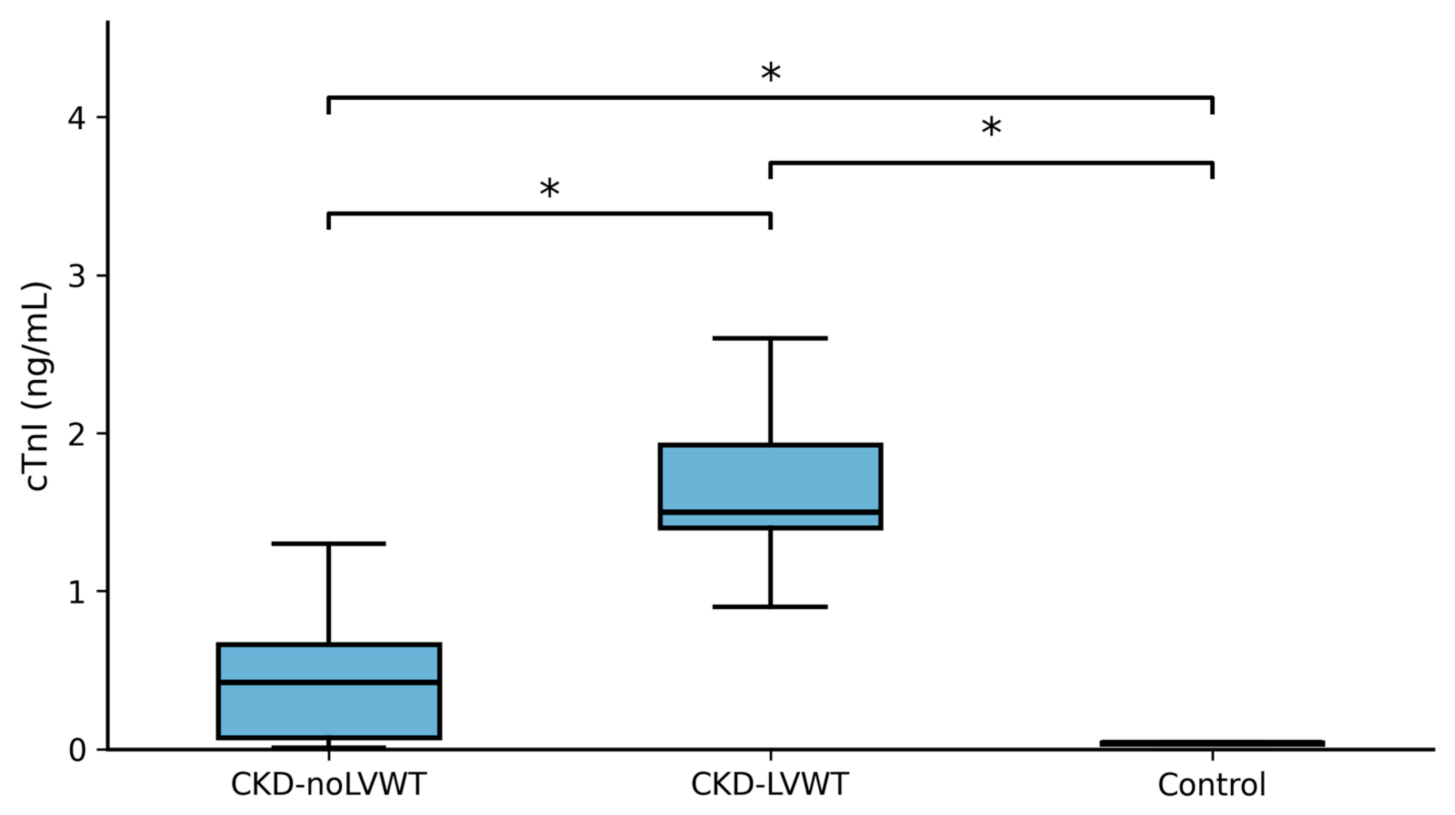
Box-and-whisker plot of serum cTnI concentrations in the CKD-noLVWT group (*n* = 12), CKD-LVWT group (*n* = 8), and control group (*n* = 11). Boxes represent the interquartile range, horizontal lines within the boxes indicate the median, and whiskers extend to the most extreme values within 1.5 times the interquartile range. The overall difference among the groups was significant (Kruskal–Wallis test, *p* < 0.001). Asterisks above the horizontal brackets indicate significant pairwise differences determined using the Conover–Iman post hoc test with Holm adjustment (CKD-LVWT versus CKD-noLVWT and CKD-LVWT versus control, adjusted *p* < 0.001; CKD-noLVWT versus control, adjusted *p* = 0.003). CKD, chronic kidney disease; LVWT, left ventricular wall thickening; cTnI, cardiac troponin I.

NT-proBNP and cTnI concentrations differed significantly among all three groups (*p* < 0.001 for both; Table 2). NT-proBNP concentrations were highest in the CKD-LVWT group (1198.10 ± 386.16), followed by the CKD-noLVWT group (517.98 ± 581.48 pmol/L) and the control group (58.64 ± 10.02 pmol/L) (Figure 1). Cardiac troponin I concentrations showed the same pattern, with values of 1.75 ± 0.77 ng/mL, 0.54 ± 0.57 ng/mL, and 0.04 ± 0.02 ng/mL in the CKD-LVWT, CKD-noLVWT, and control groups, respectively (Figure 2). The reportable range of the NT-proBNP assay was 50–1500 pmol/L. Values at the lower reportable limit were observed in 1/12 cats in the CKD-noLVWT group, 0/8 cats in the CKD-LVWT group, and 6/11 healthy controls. Values at the upper reportable limit were observed in 2/12, 4/8, and 0/11 cats, respectively.

Systolic blood pressure was significantly different among the study groups (*p* < 0.001; Table 2). SBP was highest in the CKD-LVWT group (173.46 ± 9.87 mmHg), followed by the CKD-noLVWT group (149.93 ± 13.14 mmHg), and was lowest in healthy controls (135.55 ± 4.61 mmHg). Hypertension was present in 3 of 12 cats in the CKD-noLVWT group and in 6 of 8 cats in the CKD-LVWT group; the remaining two cats in the CKD-LVWT group were normotensive.

Echocardiographic variables showed significant differences among the groups (*p* < 0.001; Table 2). LVPWd and IVSd were significantly higher in the CKD-LVWT group than in both the CKD-noLVWT and control groups. Mean LVPWd values were 5.03 ± 0.76 mm, 6.90 ± 1.07 mm, and 4.44 ± 0.48 mm in the CKD-noLVWT, CKD-LVWT, and control groups, respectively. Mean IVSd values followed a similar pattern and were 4.85 ± 0.76 mm, 6.49 ± 0.70 mm, and 4.55 ± 0.53 mm in the CKD-noLVWT, CKD-LVWT, and control groups, respectively.

LA/Ao ratio was significantly higher in the CKD -LVWT group than in the CKD-noLVWT and control groups (*p* < 0.001; Table 2). Mean LA/Ao ratios were 1.28 ± 0.15, 1.68 ± 0.10, and 1.25 ± 0.10 in the CKD-noLVWT, CKD-LVWT, and control groups, respectively. Ejection fraction was significantly higher in both CKD groups than in the control group, whereas no significant difference was detected between the two CKD groups (Holm-adjusted *p* = 0.061).

**Figure 3:**
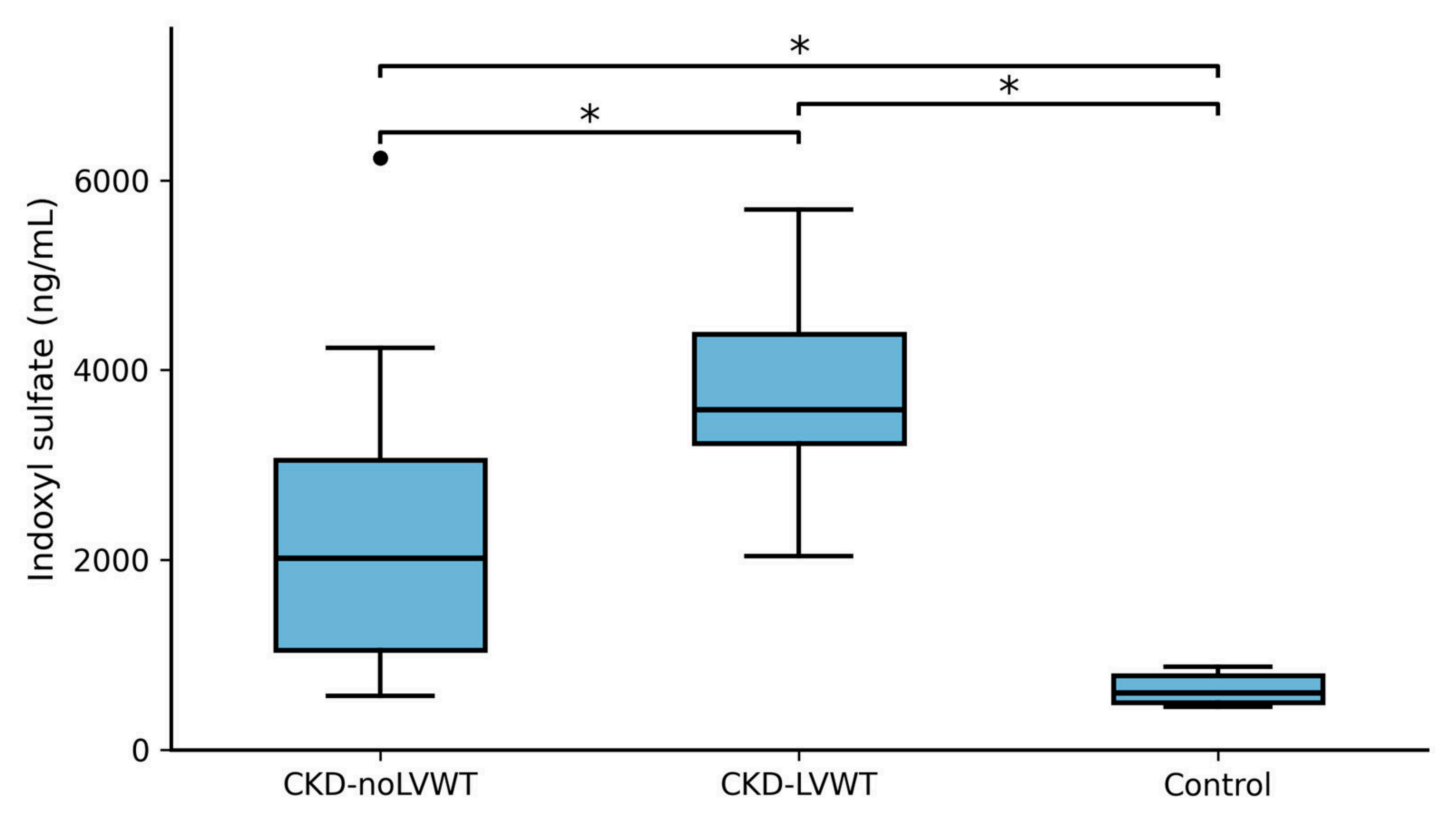
Box-and-whisker plot of plasma indoxyl sulfate concentrations in the CKD-noLVWT group (*n* = 12), CKD-LVWT group (*n* = 8), and control group (*n* = 11). Boxes represent the interquartile range, horizontal lines within the boxes indicate the median, and whiskers extend to the most extreme values within 1.5 times the interquartile range. The individual point represents an outlying value. The overall difference among the groups was statistically significant (Kruskal–Wallis test, *p* < 0.001). Asterisks above the horizontal brackets indicate significant pairwise differences determined using the Conover–Iman post hoc test with Holm adjustment (CKD-LVWT versus CKD-noLVWT, adjusted *p* = 0.008; CKD-noLVWT versus control and CKD-LVWT versus control, adjusted *p* < 0.001). CKD, chronic kidney disease; LVWT, left ventricular wall thickening.

Indoxyl sulfate concentrations differed significantly among the groups (*p* < 0.001; Table 2). Concentrations were highest in the CKD-LVWT group (3747 ± 1063 ng/mL), followed by the CKD-noLVWT group (2262 ± 1645 ng/mL) and the control group (639 ± 147 ng/mL; Figure 3).

Serum p-cresyl sulfate concentrations were significantly higher in both CKD groups than in healthy controls (overall *p* < 0.001; Table 2), but did not differ significantly between the CKD-noLVWT and CKD-LVWT groups. Mean concentrations were 45,836 ± 23,481 ng/mL, 25,736 ± 20,168 ng/mL, and 4046 ± 1496 ng/mL in the CKD-noLVWT, CKD-LVWT, and control groups, respectively.

Red blood cell count, hemoglobin concentration, and hematocrit did not differ significantly among the groups (*p* > 0.05 for all; Table 2).

Effect-size estimates and BCa bootstrap 95% confidence intervals for the principal comparisons between the CKD-LVWT and CKD-noLVWT groups are presented in Supplementary Table S2. Several confidence intervals were wide, reflecting the limited precision associated with the small group sizes.

## 4. Discussion

In the present study, serum urea, creatinine, and phosphorus concentrations were significantly higher in both CKD groups than in clinically healthy controls, consistent with reduced renal excretory function and impaired phosphorus homeostasis in advanced CKD [21,22,23]. However, none of these variables differed significantly between the CKD-noLVWT and CKD-LVWT groups. Thus, although conventional renal variables distinguished cats with CKD from clinically healthy controls, they did not differentiate between cats with and without LVWT. This finding does not exclude potential contributions from azotemia, phosphorus retention, altered volume status, or other CKD-associated metabolic disturbances to the observed differences in left ventricular structure. Rather, it suggests that these variables alone were insufficient to account for the presence of LVWT in this study population. Therefore, evaluating cardiovascular variables together with nontraditional uremic factors, including indoxyl sulfate, may provide a more complete understanding of the factors associated with LVWT in cats with CKD.

NT-proBNP and cTnI concentrations differed significantly among three groups, with the highest concentrations observed in the CKD-LVWT group, intermediate concentrations in the CKD-noLVWT group, and the lowest concentrations in clinically healthy controls. These biomarkers provide complementary information regarding myocardial stress and injury. NT-proBNP is released in response to myocardial stretch associated with pressure or volume overload, whereas cTnI is released following cardiomyocyte injury and is used as a marker of myocardial damage in cats [20,24,25]. Nevertheless, both biomarkers should be interpreted cautiously in cats with CKD because their circulating concentrations may be influenced by renal dysfunction as well as by CKD-associated cardiovascular abnormalities [24].

Previous studies have shown that NT-proBNP [26] and cTnI may be increased in cats with CKD [27,28]. Increased NT-proBNP may reflect reduced renal clearance, mild volume expansion, increased myocardial wall stress, or early cardiac involvement [25]. Increased cTnI is more likely to reflect subclinical cardiomyocyte injury, although reduced elimination of circulating troponin fragments and CKD-associated microvascular, inflammatory, and metabolic changes may also contribute [24,29,30].

In the present study, both cardiac biomarkers were higher in the CKD–noLVWT group than in clinically healthy controls, suggesting that renal dysfunction and/or myocardial stress or injury may influence their circulating concentrations even in the absence of echocardiographically defined LVWT. However, NT-proBNP and cTnI concentrations were substantially higher in the CKD-LVWT group, despite similar serum urea, creatinine, and phosphorus concentrations in the two CKD groups. Cats in the CKD-LVWT group also had higher systolic blood pressure. Taken together, these findings indicate that the greater biomarker elevations in this group cannot be attributed to differences in conventional renal variables alone. They may reflect the combined influence of systolic hypertension, increased myocardial stress or injury, and other CKD-related factors. Therefore, NT-proBNP and cTnI should be interpreted alongside renal variables, systolic blood pressure, and echocardiographic findings in cats with CKD.

Systolic hypertension is a common complication of feline CKD and may contribute to left ventricular hypertrophy by increasing left ventricular afterload [6,20]. In the present study, systolic blood pressure and LA/Ao ratio were higher in the CKD-LVWT group than in the other groups. Sustained pressure overload can increase myocardial wall stress and promote concentric left ventricular hypertrophy. Thus, systemic hypertension is a plausible contributor to the LVWT observed in the CKD-LVWT group.

However, hypertension and LVWT did not completely overlap. Two of the eight cats in the CKD-LVWT group were normotensive, whereas three of the 12 in the CKD-noLVWT group were hypertensive. The absence of LVWT in some hypertensive cats may reflect differences in the duration of hypertension or in individual myocardial susceptibility. Conversely, the presence of LVWT in normotensive cats suggests that factors other than pressure overload may also be involved. Although cats diagnosed with primary cardiomyopathy or other cardiac diseases were excluded, occult primary hypertrophic cardiomyopathy could not be completely ruled out. Indoxyl sulfate may represent another potential factor associated with LVWT. Experimental studies have linked this protein-bound uremic toxin to endothelial dysfunction, vascular stiffness, oxidative stress, inflammation, cardiomyocyte hypertrophy, and myocardial fibrosis [14,16]. The higher plasma indoxyl sulfate concentration observed in the CKD-LVWT group is consistent with a possible contribution of uremia-associated mechanisms. Nevertheless, the observational design of this study does not allow the relative contributions of blood pressure and indoxyl sulfate to be determined. LVWT in cats with CKD may therefore reflect a combination of hemodynamic and non-hemodynamic processes.

In the present study, the term left ventricular wall thickening was used instead of cardiac remodeling because progressive structural change was not documented over an extended follow-up period. The three echocardiographic examinations performed within one month were intended to confirm the persistence of wall thickening and minimize the influence of measurement variability or transient changes, rather than to demonstrate a longitudinal remodeling process. The higher systolic blood pressure in the CKD-LVWT group is consistent with a possible contribution of chronic pressure overload. However, these findings do not establish whether LVWT was attributable to hypertension, uremic factors, an interaction between these factors, or occult primary cardiomyopathy.

The two protein-bound uremic toxins showed different patterns across the study groups. Plasma IS concentrations were highest in the CKD-LVWT group, followed by the CKD-noLVWT group and clinically healthy controls. Although serum urea, creatinine, and phosphorus concentrations did not differ significantly between the two CKD groups, plasma IS concentrations were significantly higher in the CKD-LVWT group. In contrast, serum PCS concentrations were higher in both CKD groups than in clinically healthy controls but did not differ significantly between the CKD-noLVWT and CKD-LVWT groups. Thus, only IS differed between the CKD groups categorized according to LVWT status. This unadjusted association should be interpreted cautiously because blood pressure, CKD stage, treatment, and other potential confounding variables were not accounted for in a multivariable model. Although IS and PCS are both gut-derived, protein-bound uremic retention solutes, they arise from different metabolic precursors and may have distinct biological effects. IS is derived primarily from tryptophan metabolism, whereas PCS is derived from tyrosine and phenylalanine metabolism [13].

Experimental studies provide biological plausibility for a relationship between IS and LVWT. Lekawanvijit et al. showed that IS increased collagen synthesis in cardiac fibroblasts, induced cardiomyocyte hypertrophy, and activated pro-inflammatory pathways involving p38, p42/44 MAPK, and NF-κB [31]. Kishimoto et al. subsequently reported that IS promoted left ventricular hypertrophy through activation of the aryl hydrocarbon receptor–FGF23–FGFR4 signaling pathway in cultured cardiomyocytes and a murine model of CKD [32]. Against this experimental background, plasma IS concentrations were significantly higher in the CKD-LVWT group, despite similar serum urea, creatinine, and phosphorus concentrations in the two CKD groups. This pattern suggests that IS may be associated with LVWT beyond the information provided by conventional renal variables. However, systolic blood pressure was also higher in the CKD-LVWT group, and the absence of multivariable adjustment prevented us from determining whether the association with IS was independent of blood pressure, CKD stage, treatment, or other potential confounders. Accordingly, the present findings should be regarded as evidence of an exploratory, unadjusted association rather than evidence that IS independently or causally contributes to LVWT in cats with CKD.

In a hypertensive rat model, Yisireyili et al. showed that IS aggravated cardiac fibrosis and cardiomyocyte hypertrophy and increased the expression of profibrotic and oxidative stress-related markers [15]. These changes occurred without significant differences in blood pressure or renal function between the hypertensive groups, suggesting an effect that was not attributable solely to greater hemodynamic load or more severe renal dysfunction. In contrast, cats in the CKD-LVWT group in the present study had both higher systolic blood pressure and higher plasma IS concentrations. Therefore, although the findings are compatible with the involvement of both pressure overload and IS-associated mechanisms in the presence of LVWT, their relative contributions and any interaction between them cannot be determined from these observational data.

The present study has several limitations. The sample size was small, particularly in the CKD-LVWT group, which limited statistical power, precluded reliable multivariable adjustment, and may restrict the generalizability of the findings. This limited precision was reflected in the wide confidence intervals around several effect-size estimates. Consequently, the subgroup comparisons may be sensitive to individual observations and should be interpreted cautiously as exploratory findings rather than stable estimates of population-level effects. Although the cats were enrolled prospectively, the primary analysis was cross-sectional. Consequently, the temporal relationship between indoxyl sulfate accumulation and the development or progression of LVWT could not be determined. The potential confounding effects of systolic blood pressure, CKD stage, treatment, and other variables could not be adequately evaluated. Antihypertensive treatment and adherence represent particularly important potential confounders. The hypertensive cats in the CKD-noLVWT group received amlodipine inconsistently, whereas the prescribed amlodipine was never administered to the six hypertensive cats in the CKD-LVWT group. Consequently, absent or inadequate antihypertensive exposure may have contributed to persistently elevated SBP and cumulative pressure load. Because treatment exposure and adherence could not be incorporated into a multivariable analysis, their effects could not be separated from those of SBP, IS, or other CKD-associated factors. Therefore, the observed associations should be interpreted as exploratory and hypothesis-generating rather than causal.

Only total IS and PCS concentrations were measured. Free toxin fractions and factors that may affect protein binding, renal tubular elimination, dietary exposure, or gut microbial toxin production were not assessed. Proteinuria substaging was not performed, and renal ultrasonographic findings were not systematically available for all cats. Therefore, the potential effects of proteinuria and structural renal abnormalities on toxin concentrations and LVWT could not be evaluated.

LVWT was defined as IVSd and/or LVPWd ≥ 6 mm in at least two of three echocardiographic examinations performed within one month. Although these repeated assessments confirmed the persistence of wall thickening, they did not establish its underlying cause or demonstrate longitudinal remodeling. Body weight did not differ significantly among the groups; nevertheless, the fixed threshold of ≥6 mm may not fully account for individual body-size-related variation in LV wall thickness. In addition, Teichholz-derived EF relies on linear LV dimensions and geometric assumptions and may not fully represent global systolic function when LV geometry is altered. Myocardial fibrosis and cardiomyocyte hypertrophy were not evaluated histopathologically. Furthermore, although cats with a previous diagnosis of primary cardiomyopathy or overt echocardiographic findings suggestive of primary cardiomyopathy were excluded, occult or early-stage primary hypertrophic cardiomyopathy could not be ruled out. The observed LVWT therefore cannot be attributed exclusively to systemic hypertension, CKD, or uremic toxin accumulation.

Finally, renal dysfunction may influence circulating NT-proBNP and cTnI concentrations, thereby complicating their interpretation in cats with CKD. Although these cardiac biomarkers were evaluated alongside systolic blood pressure and echocardiographic findings, their clinical and prognostic significance in this population remains uncertain. Larger longitudinal studies incorporating serial biomarker and echocardiographic assessments are needed to determine whether changes in NT-proBNP, cTnI, and indoxyl sulfate precede or accompany the development and progression of LVWT in cats with CKD.

## 5. Conclusions

Left ventricular wall thickening in cats with chronic kidney disease may involve multiple factors that are not fully captured by conventional renal variables alone. Although serum urea, creatinine, and phosphorus concentrations were similarly increased in both CKD groups, cats in the CKD-LVWT group had higher systolic blood pressure, cardiac biomarker concentrations, and plasma indoxyl sulfate concentrations. In contrast, serum p-cresyl sulfate concentrations were increased in both CKD groups but did not differ according to LVWT status. Within this study population, higher plasma indoxyl sulfate concentrations were associated with LVWT. However, the marked elevation in systolic blood pressure in the CKD-LVWT group represents a major potential source of confounding because pressure overload may itself contribute to LVWT. As reliable multivariable adjustment could not be performed, the observed association between indoxyl sulfate and LVWT cannot be considered independent of blood pressure, CKD stage, treatment, or other potential confounders and should not be interpreted as causal. NT-proBNP and cTnI should therefore be interpreted in conjunction with renal variables, systolic blood pressure, and echocardiographic findings. Larger longitudinal studies incorporating appropriate multivariable analyses are needed to clarify the temporal and independent relationships among indoxyl sulfate accumulation, blood pressure, and LVWT in cats with CKD.

## Figures and Tables

**Table 1:** Descriptive statistics regarding demographic characteristics of the study population.

			Groups			
			CKD-noLVWT (*n* = 12)	CKD-LVWT (*n* = 8)	Control (*n* = 11)	*p*
**Age (years)**		Mean ± SD	8.50 ± 1.68	9.00 ± 1.69	8.82 ± 1.83	*p* = 0.934 ^x^
		Median	8.00	8.50	9	
**Body weight (kg)**		Mean ± SD	4.06 ± 0.48	3.90 ± 0.46	4.16 ± 0.55	*p* = 0.532 ^x^
		Median	4.10	3.80	4.00	
**Sex**	Female	n (%)	5 (41.7%)	3 (37.5%)	6 (54.5%)	*p* =0.726 ^y^
	Male	n (%)	7 (58.3%)	5 (62.5%)	5 (45.5%)	
**Stage 3**		n (%)	4 (33.3)	2 (25.0)	N/A	*p* =1.000 ^z^
**Stage 4**		n (%)	8 (66.7)	6 (75.0)	N/A	

Data are presented as the mean ± standard deviation (SD), median, or number and percentage [n (%)], as appropriate. Superscript indicates the statistical tests used: x, one-way analysis of variance, y indicates Pearson’s chi-square test, and z indicates Fisher’s exact test. CKD, chronic kidney disease; LVWT, left ventricular wall thickening; CKD-noLVWT, CKD without LVWT; CKD-LVWT, CKD with LVWT; N/A, not applicable.

**Table 2:** Hematological, serum biochemical, cardiac biomarker, systolic blood pressure, echocardiographic, and uremic toxin variables in CKD-noLVWT, CKD-LVWT, and clinically healthy controls.

	RI	CKD-noLVWT (*n* = 12)		CKD-LVWT (*n* = 8)		Control (*n* = 11)		*p*
		Mean ± SD	Median (Range)	Mean ± SD	Median (Range)	Mean ± SD	Median (Range)	
Urea (mg/dL)	42.8–64.2	277.19 ± 190.80 ^a^	231.33 (110.00–715.50)	280.21 ± 109.80 ^a^	251.65 (164.00–482.00)	48.66 ± 10.86 ^b^	53.00 (30.00–60.00)	<0.001 ^y^
Creatinine (mg/dL)	0.8–1.6	6.46 ± 2.58 ^a^	6.12 (2.94–10.70)	7.67 ± 4.82 ^a^	6.63 (3.26–19.00)	1.09 ± 0.17 ^b^	1.10 (0.80–1.32)	<0.001 ^y^
Phosphorus (mg/dL)	4.0–7.3	10.16 ± 4.92 ^a^	8.18 (4.40–19.70)	10.66 ± 4.13 ^a^	10.80 (4.42–18.00)	4.61 ± 0.49 ^b^	4.70 (3.64–5.30)	<0.001 ^y^
NT-proBNP (pmol/L)	<100	517.98 ± 581.48 ^b^	213.35 (50.00–1500.00)	1198.10 ± 386.16 ^a^	1350.00 (481.10–1500.00)	58.64 ± 10.02 ^c^	50.00 (50.00–70.00)	<0.001 ^y^
cTnI (ng/mL)	<0.18 ng/mL	0.54 ± 0.57 ^b^	0.42 (0.01–1.90)	1.75 ± 0.77 ^a^	1.50 (0.90–3.20)	0.04 ± 0.02 ^c^	0.04 (0.01–0.09)	<0.001 ^y^
LVPWd (mm)	<6.0 ^†^	5.03 ± 0.76 ^b^	5.30 (3.40–5.80)	6.90 ± 1.07 ^a^	6.50 (5.80–9.00)	4.44 ± 0.48 ^b^	4.20 (3.90–5.40)	<0.001 ^x^
IVSd (mm)	<6.0 ^†^	4.85 ± 0.76 ^b^	5.10 (3.50–5.80)	6.49 ± 0.70 ^a^	6.25 (6.10–8.20)	4.55 ± 0.53 ^b^	4.40 (3.90–5.70)	<0.001 ^y^
EF (%)	Laboratory-/method-specific	85.33 ± 3.70 ^a^	87.00 (78.00–89.00)	89.38 ± 6.12 ^a^	92.50 (77.00–94.00)	77.45 ± 3.4 ^bc^	79.00 (70.00–82.00)	<0.001 ^y^
SBP (mmHg)	<140; hypertension ≥ 160 ^‡^	149.93 ± 13.14 ^b^	146.60 (136.00–178.00)	173.46 ± 9.87 ^a^	174.10 (157.80–184.00)	135.55 ± 4.61 ^c^	137.00 (128.00–142.00)	<0.001 ^y^
LA/Ao	<1.6	1.28 ± 0.15 ^b^	1.30 (1.10–1.50)	1.68 ± 0.10 ^a^	1.70 (1.50–1.80)	1.25 ± 0.10 ^b^	1.20 (1.10–1.40)	<0.001 ^x^
Indoxyl sulfate (ng/mL)	Not established	2262 ± 1645 ^b^	2019 (567–6231)	3747 ± 1063 ^a^	3582 (2036–5691)	639 ± 147 ^c^	597 (455–876)	<0.001 ^y^
p-cresyl sulfate (ng/mL)	Not established	45836 ± 23481 ^a^	50992 (3624–72524)	25736 ± 20168 ^a^	22032 (5338–65690)	4046 ± 1496 ^b^	3622 (2234–7658)	<0.001 ^y^
RBC (×10^6^/µL)	4.60–10.20	6.98 ± 2.19 ^a^	7.19 (4.01–10.02)	6.90 ± 2.23 ^a^	7.44 (3.10–9.67)	8.75 ± 1.49 ^a^	9.22 (5.57–10.20)	0.068 ^x^
HGB (g/dL)	8.50–15.30	10.85 ± 4.11 ^a^	9.95 (4.90–17.00)	9.98 ± 3.30 ^a^	10.85 (5.00–15.00)	12.41 ± 2.04 ^a^	13.00 (9.30–14.90)	0.272 ^x^
HCT (%)	26.00–47.00	29.73 ± 9.66 ^a^	28.45 (15.10–43.70)	30.36 ± 9.76 ^a^	32.40 (17.60–42.30)	36.36 ± 4.61 ^a^	36.00 (28.90–42.00)	0.213 ^y^

Values are presented as mean ± standard deviation (SD) and median (range, minimum–maximum). Within each row, values with different superscript lowercase letters differ significantly between groups (*p* < 0.05), whereas values sharing the same letter are not significantly different. Superscript x indicates variables analyzed using one-way analysis of variance followed by Tukey’s post hoc test. Superscript y indicates variables analyzed using the Kruskal–Wallis test, Conover–Iman post hoc test with Holm adjustment for multiple comparisons. RI denotes the laboratory reference interval or manufacturer-provided clinical reference limit, where available. Manufacturer-provided clinical threshold for the Vcheck Feline TnI assay. ^†^ Threshold used in the present study to define LVWT; values between 5 and 6 mm may be considered equivocal depending on clinical context. ^‡^ SBP < 140 mmHg represents the normotensive risk category, whereas persistent SBP ≥ 160 mmHg is consistent with hypertension. Laboratory-specific reference intervals were used for hematological and biochemical variables. Validated feline reference intervals have not been established for indoxyl sulfate and p-cresyl sulfate. CKD, chronic kidney disease; LVWT, left ventricular wall thickening; CKD-noLVWT, CKD without LVWT; CKD-LVWT, CKD with LVWT; NT-proBNP, N-terminal pro-B-type natriuretic peptide; cTnI, cardiac troponin I; LVPWd, left ventricular posterior wall thickness in diastole; IVSd, interventricular septal thickness in diastole; EF, ejection fraction; SBP, systolic blood pressure; LA/Ao, left atrial-to-aortic root ratio; RBC, red blood cell count; HGB, hemoglobin; HCT, hematocrit.

## Data Availability

The original contributions presented in this study are included in the article/Supplementary Material. Further inquiries can be directed to the corresponding author(s).

## Supplementary Materials

The following supporting information can be downloaded at: https://www.mdpi.com/article/10.3390/vetsci13090989/s1. Table S1: Analytical validation characteristics of the HPLC method used for the determination of indoxyl sulfate and p-cresyl sulfate; Figure S1: Representative HPLC chromatograms of indoxyl sulfate and p-cresyl sulfate in standard solutions and feline samples; Table S2: Effect-size estimates and BCa bootstrap 95% confidence intervals for the principal comparisons between the CKD-LVWT and CKD-noLVWT groups.

## Abbreviations

The following abbreviations are used in this manuscript:

ACVIM	American College of Veterinary Internal Medicine
AhR	Aryl hydrocarbon receptor
CKD	Chronic kidney disease
cTnI	Cardiac troponin I
EF	Ejection fraction
FGF23	Fibroblast growth factor 23
FGFR4	Fibroblast growth factor receptor 4
FS	Fractional shortening
HPLC	High-performance liquid chromatography
IRIS	International Renal Interest Society
IS	Indoxyl sulfate
IVSd	Interventricular septal thickness in diastole
LA/Ao	Left atrial-to-aortic root ratio
LVWT	Left ventricular wall thickening
LVPWd	Left ventricular posterior wall thickness in diastole
MAPK	Mitogen-activated protein kinase
NF-κB	Nuclear factor kappa B
NT-proBNP	N-terminal pro-B-type natriuretic peptide
PCS	p-Cresyl sulfate
SBP	Systolic blood pressure
USG	Urine specific gravity
