# Supplementary material for: Indoxyl Sulfate, Cardiac Biomarkers, and Left Ventricular Wall Thickening in Cats with Chronic Kidney Disease
Source: Vet Sci. 2026 Sep 19;13(9):989. doi: 10.3390/vetsci13090989 (PMC13611987; doi:10.3390/vetsci13090989)
Supplement: Supplementary file 1 [file vetsci-13-00989-s001.zip › vetsci-4502742-supplementary.pdf]

Table S1. Analytical validation characteristics of the HPLC method used for the determination of indoxyl sulfate and p-cresyl sulfate.

Validation parameter	IS	PCS
Validated calibration range (µM)	2-50	5-500
Coefficient of determination (R^2)	0.9987	0.9988
LOD (µM)	1.0	0.5
LLOQ (µM)	2.0	5.0
Intra-day precision (%CV)	2-8	3-9
Inter-day precision (%CV)	3-10	4-11
Recovery (%)	92-105	90-105

Abbreviations: IS, indoxyl sulfate; PCS, p- cresyl sulfate; LOD, limit of detection; LLOQ, lower limit of quantification; CV, coefficient of variation. Precision and recovery were evaluated at representative low, medium and high concentration levels.

Table S2. Effect sizes and 95% confidence intervals for the principal comparisons between the CKD-LVWT and CKD-noLVWT groups.

Variable	Cliff's δ	BCa bootstrap 95% CI	Holm-adjusted *P* value
Indoxyl sulfate	0.625	0.042–0.917	0.008
p-Cresyl sulfate	−0.500	−0.854–0.063	0.068
NT-proBNP	0.688	0.208–0.917	0.003
cTnI	0.833	0.292–1.000	<0.001
Systolic blood pressure	0.823	0.354–0.979	0.001

Effect sizes are reported as Cliff's delta (δ) for comparisons between the CKD-LVWT and CKD-noLVWT groups. Positive values indicate higher measurements in the CKD-LVWT group, whereas negative values indicate higher measurements in the CKD-noLVWT group. Confidence intervals were calculated using the bias-corrected and accelerated (BCa) bootstrap method with 1,000,000 resamples. Adjusted *P* values were obtained using the Conover–Iman post hoc test with Holm adjustment for multiple comparisons. CKD, chronic kidney disease; LVWT, left ventricular wall thickening; NT-proBNP, N-terminal pro-B-type natriuretic peptide; cTnI, cardiac troponin I; CI, confidence interval.

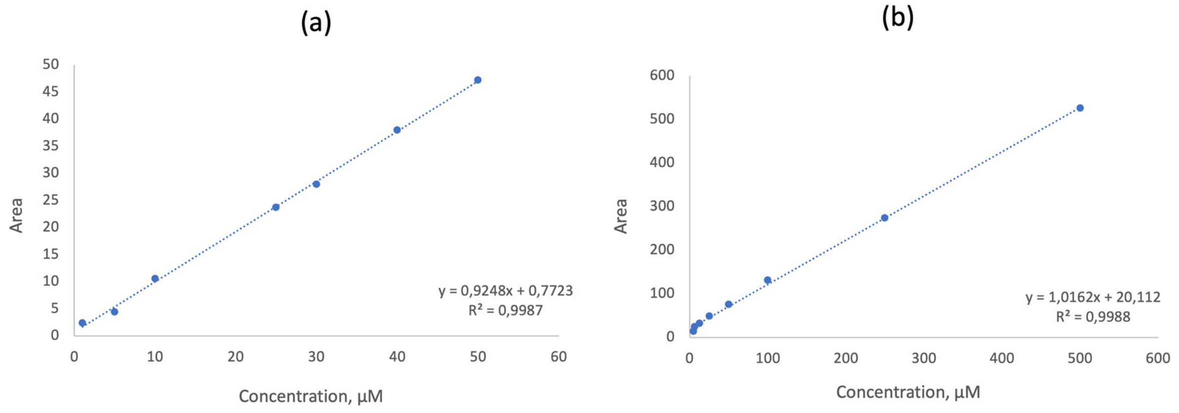

Figure S1. Calibration curves for the HPLC determination of indoxyl sulfate (IS) and p- cresyl sulfate (PCS). (a) Calibration curve for IS over the validated concentration range of 2-50 µM. (b) Calibration curve for PCS over the validated concentration range of 5-500 µM. Peak area was plotted against analyte concentration.
